# MOTiFS: Monte Carlo Tree Search Based Feature Selection

**DOI:** 10.3390/e20050385

**Published:** 2018-05-20

**Authors:** Muhammad Umar Chaudhry, Jee-Hyong Lee

**Affiliations:** Department of Electrical and Computer Engineering, Sungkyunkwan University, Suwon 16419, Korea

**Keywords:** feature selection, Monte Carlo Tree Search (MCTS), heuristic feature selection, dimensionality reduction, wrapper, MOTiFS

## Abstract

Given the increasing size and complexity of datasets needed to train machine learning algorithms, it is necessary to reduce the number of features required to achieve high classification accuracy. This paper presents a novel and efficient approach based on the Monte Carlo Tree Search (MCTS) to find the optimal feature subset through the feature space. The algorithm searches for the best feature subset by combining the benefits of tree search with random sampling. Starting from an empty node, the tree is incrementally built by adding nodes representing the inclusion or exclusion of the features in the feature space. Every iteration leads to a feature subset following the *tree* and *default* policies. The accuracy of the classifier on the feature subset is used as the reward and propagated backwards to update the tree. Finally, the subset with the highest reward is chosen as the best feature subset. The efficiency and effectiveness of the proposed method is validated by experimenting on many benchmark datasets. The results are also compared with significant methods in the literature, which demonstrates the superiority of the proposed method.

## 1. Introduction

In the current era of information overload, the size of datasets is growing extensively. This leads to the high dimensional datasets containing many redundant and irrelevant features, resulting in computationally expensive analysis and less accurate predictive modeling [1,2,3]. Feature selection comes to the rescue and aids in reducing dimensions. The feature selection algorithm looks for the optimal or most informative features by putting aside the redundant and irrelevant features, retaining accurate information and data structures where possible, resulting in efficient and more accurate predictive models. Feature selection has been studied for decades in various fields including machine learning [4,5,6], statistics [7,8], pattern recognition [9,10,11], and data mining [12,13].

**Figure 1:**
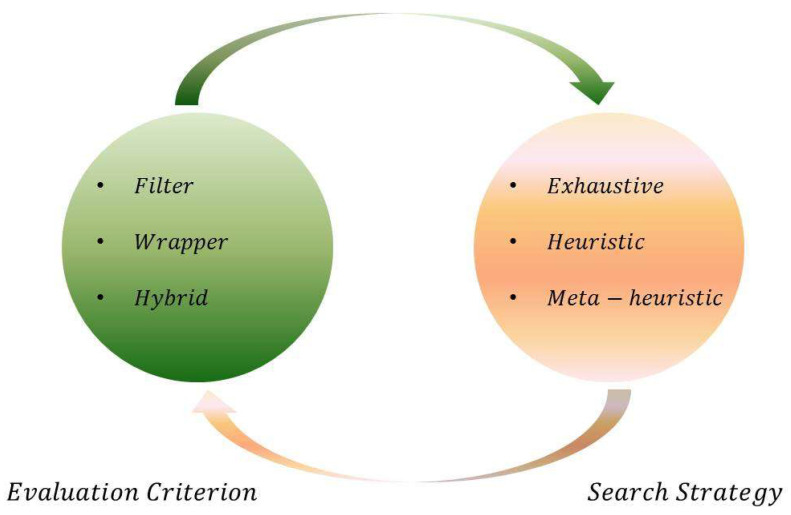
Key aspects of feature selection.

When addressing the feature selection problem, there are two key aspects: *search strategy* and *evaluation criterion*. An efficient *search strategy* finds the best candidate subsets rather trying each and every possible subset, thus reducing the time complexity. A good *evaluation criterion* judges the goodness of candidate subsets and identifies the best one among them, thus improving performance in terms of accuracy. Based on the *evaluation criterion*, feature selection approaches are mainly classified as filter, wrapper, or hybrid approaches. In terms of the *search strategy* (ignoring *evaluation criteria*), feature selection algorithms can be classified into exhaustive search, heuristic search, or meta-heuristic search-based methods. Figure 1 shows the key aspects and classifications of feature selection methods.

Meta-heuristic approaches, often referred as Evolutionary Algorithms (EA), have recently gained much attention in feature selection [14]. Meta-heuristic algorithms dig the search space by keeping the good solutions and improving them (exploitation), as well as looking for the new ones in other areas through the search space (exploration). Examples of Evolutionary Algorithms are the Genetic Algorithm (GA) [15,16], Ant Colony Optimization (ACO) [17,18], Particle Swarm Optimization (PSO) [19,20,21], Multi-Objective Evolutionary Algorithms [22,23,24] and Bat Algorithms [25,26]. The use of these approaches is still in infancy stages with a debate on which approach is better than the others. Although the performance of meta-heuristic algorithms is pretty useful compared to traditional heuristic approaches, they are complex in that they need to be fine-tuned on many hyper parameters and need enough time to achieve convergence [27]. The tradeoff between the computational feasibility, model complexity and optimal features selection is still an unsolved puzzle among all these methods [14]. Therefore, vast room for improvement is available and new algorithms are immensely needed to overcome such issues, which can *efficiently* achieve *high accuracy* with *less model complexity*.

In this paper, we present a novel approach for feature selection which combines the robustness and dynamicity of Monte Carlo Tree Search (MCTS) with the accuracy of wrapper methods. We employ MCTS as an efficient search strategy within wrapper framework developing the efficient and effective algorithm, named as MOTiFS (Monte carlO Tree Search Based Feature Selection). MCTS is a search strategy which finds the optimal solutions probabilistically by using lightweight random simulations [28]. It takes random samples in the search space and builds the search tree accordingly. Currently, MCTS is successfully being deployed in games with huge search space [29]. However, its effectiveness is not well explored for feature selection problems, which is the major motivation of this study.

The proposed algorithm, MOTiFS, starts with an empty tree node, meaning no feature has been selected. The tree is then incrementally built by adding nodes one by one representing either of the two corresponding feature states: a feature is selected or not selected. Every iteration leads to the generation of a feature subset following the *tree* and *default* policies. The *tree* policy not only exploits the expanded feature space by searching for the features which have already shown good performance in the previous iterations, but also explores the new features by expanding the tree incrementally. The *default* policy, then, induces randomness by choosing the features randomly from the remaining set of yet unexpanded features. This perfect blend of tree search with random sampling accelerates the process and provides the opportunity to generate the best feature subset in a few iterations, even if the search tree is not fully expanded. MOTiFS uses the classification accuracy as a goodness of the current feature subset as well as the reward for the current iteration. The search tree is then updated by propagating the reward backwards through the selected nodes. Finally, the feature subset with highest accuracy is chosen as a best feature subset. For experimental purposes, the K-Nearest Neighbor classifier is employed as a reward function. MOTiFS is tested on 25 real-world datasets and the promising results prove its validity. The comparison with latest and state-of-the art methods shows the superiority of MOTiFS and serves as a proof of concept. The main contributions of this study are listed below:

- The novel feature selection algorithm, MOTiFS, is proposed which combines the robustness of MCTS with the accuracy of wrapper methods.
- MOTiFS searches through the feature space efficiently and find the best feature subset within a few iterations, relatively.
- Only two hyper-parameters, *scaling factor* and *termination criteria*, are required to be tuned, making MOTiFS simple and flexible to handle.
- MOTiFS is tested on 25 benchmark datasets and results are also compared with other established methods. The promising results demonstrate the superiority of MOTiFS.

The rest of the paper is organized as follows. The review of the literature is provided in Section 2. Section 3 provides the necessary background for the proposed method. Section 4 presents the demonstration of the proposed method (MOTiFS). The results and experimental details are presented in Section 5. Finally, the conclusions and future research directions are discussed in Section 6.

## 2. Literature Review

The key aspects of feature selection are illustrated above in Figure 1. This section presents a brief overview of various feature selection methods.

Filter methods are independent of the specific classification algorithm. They use the inherent properties, like distance and information gain, of the dataset and measure the importance of each feature with respect to the class label and rank them [30,31,32]. Filter-based methods are fast enough and can be used with any classification algorithm, but there is a major drawback in that they show a lower performance in terms of classification accuracy. In wrapper methods, the classification algorithm is directly related in a way that the accuracy of the classifier serves as a measure of goodness of the candidate feature subsets [33,34,35]. They are computationally expensive because they run the classifier repeatedly but deliver high accuracy compared to filter methods. Hybrid methods integrate the filter and wrapper methods in order to take advantage of both types [36,37]. Such methods use the independent metric and a learning algorithm in order to measure the goodness of each candidate feature subset in the search space.

Irrespective of the evaluation criterion, feature selection methods fall into one of the following search strategies: exhaustive, heuristic or meta-heuristic. In earlier literature, a few attempts at feature selection have been made, involving exhaustive searches [38]. However, applying an exhaustive search on datasets with many features is practically impossible due to the complexity involved, so they are seldom used. Hence, researchers have adopted heuristic search strategies, like greedy hill climbing and best first search, which use some heuristics to reach the goal rather traversing the whole search space [30,39]. Greedy hill climbing approaches include SFS (Sequential Forward Selection), SBS (Sequential Backward Selection) and bidirectional search algorithms. They look for the relevant features by evaluating all local changes in a search space. However, the major drawback associated with such algorithms is that whenever a positive change occurs—either a feature is added to the selected set in SFS or deleted from the selected set in SBF—this feature does not get a chance to be re-evaluated, and it becomes highly probable that the algorithm will deviate from optimality. Such a problem is referred as the *nesting effect* [14]. In efforts to tackle this major issue, researchers came up with some useful algorithms like SFFS (Sequential Forward Floating Selection) and SBFS (Sequential Backward Floating Selection).

Recently, meta-heuristic approaches like the Genetic Algorithm (GA) [15,16], Ant Colony Optimization (ACO) [17,18], Particle Swarm Optimization (PSO) [19,20,21], Multi-Objective Evolutionary Algorithms [22,23,24] and Bat Algorithms [25,26] have gained much attention [14]. However, the involvement of too many hyper parameters makes tuning the models for optimized performance too complex [27]. For example, in GAs, a sufficient population size is required with high enough generations to obtain the desired results. Obviously, this leads GAs to be computationally expensive. Also, many parameters are involved in GAs, like population size, number of generations, crossover probability, permutation probability, etc., which makes it more challenging to find the suitable model for effective feature selection. One earlier attempt using MCTS in feature selection is found in [40]. The method proposed in [40] maps the feature selection as an exhaustive search tree and, therefore, has a huge branching factor and is computationally very expansive with unacceptable bounds.

In this study, we proposed a novel feature selection algorithm based on MCTS and wrapper methods. We define the feature selection tree in a novel and incremental fashion, where exploration and exploitation are well balanced within limited computational bounds. The extensive experimentation on many benchmark datasets and comparison with state-of-the-art methods demonstrates the validity of the proposed method.

## 3. Background

This section presents the background concepts used in the proposed method.

### 3.1. Working Procedure of Monte Carlo Tree Search (MCTS)

MCTS is a heuristic search method which uses lightweight random simulations to reach a goal state [28]. Each MCTS iteration consists of four sequential steps: selection, expansion, simulation and backpropagation.

1. *Selection*: Starting from the root node, the algorithm traverses the tree by selecting nodes with the highest approximated values, until a non-terminal node with unexpanded children is reached.
2. *Expansion*: A new child node is added to expand the tree, according to the available set of actions.
3. *Simulation*: From the new child node, a random simulation is performed until the terminal node is reached, to approximate the reward.
4. *Backpropagation*: The simulation result (reward) is backpropagated through the selected nodes to update the tree.

The *selection* and *expansion* steps are performed using the *tree policy*, whereas the *simulation* step is performed with the *default policy*.

### 3.2. Upper Confidence Bounds for Trees (UCT) Algorithm

The *tree policy* uses the Upper Confidence Bounds for Trees (UCT) algorithm for node selection. The value of each node is approximated using the UCT algorithm, as shown in Equation (1). The *tree policy* then selects the nodes at each level which have the highest approximated values. This maintains a balance between exploiting the good solutions and exploring the new ones.

(1) $$UCT_{v}=\;\frac{W_{v}}{N_{v}}+\;C\;\times \sqrt{\frac{2\;\times ln\left(N_{p}\right)}{N_{v}}},$$

where, $N_{v}$ and $N_{p}$ represents number of times nodes v and its parent p are visited, respectively. $W_{v}$ represents the number of wining simulations (considering a games perspective) at node v. C is the exploration constant.

## 4. MOTiFS (Monte Carlo Tree Search Based Feature Selection)

**Figure 2:**
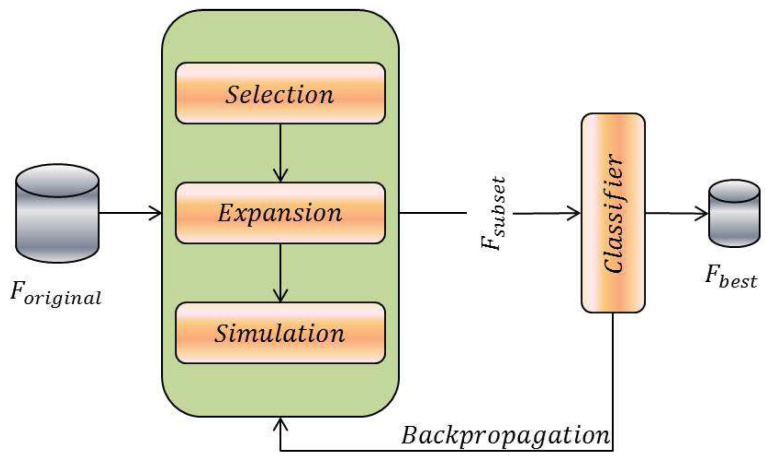
The proposed method, MOTiFS (Monte Carlo Tree Search Based Feature Selection).

The Monte Carlo Tree Search (MCTS) is used as a search strategy within a wrapper framework to develop a novel approach for feature selection. Using the efficient and meta-heuristic approach of MCTS and the predictive accuracy of the wrapper method, the goal is to find the best feature subset to give maximum classification accuracy. Figure 2 shows the depiction of the proposed method.

The preliminary step is to map the feature selection problem into some sort of game tree. In feature selection, either a feature is selected or not selected in a feature subset, and represented by 1 or 0 at the corresponding feature position in a total set of feature space. Using this intuition, we map the problem as a single player game where the goal is to select best possible features with the maximum accumulative reward. MOTiFS constructs a special tree where each node represents either of the two corresponding feature states: a feature is selected or not selected. The definition for the feature selection tree is provided below:

**Definition** **1.** *For a feature set, $F=\left\{f_{1},\;f_{2},\;\ldots ,\;f_{i},\;\ldots ,\;f_{n}\right\}$, the feature selection tree is a tree satisfying the following conditions:*

1. *The root is $\emptyset_{0}$, which means no features are selected.*
2. *Any node at level*i−1*has two children,*$f_{i}$*and*$\emptyset_{i}$*, where*0<i<n.

Where, nodes $f_{i}$ and $\emptyset_{i}$ represent the inclusion or exclusion of the corresponding feature, $f_{i}$, in the feature subset, respectively. Any path from the root node to one of the leaves represents a feature subset. So, the goal is to find a path which gives the best reward (accuracy). We use MCTS for tree construction and traversal, and finally choose the path (feature subset) with best accuracy.

**Figure 3:**
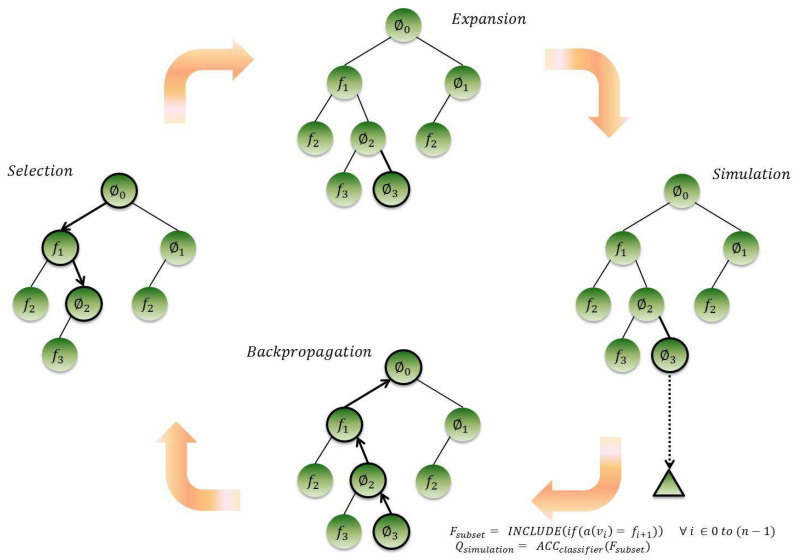
Feature selection tree and search procedure of MOTiFS.

The search starts with an empty root node and incrementally builds the tree by adding nodes representing features states, one by one, with a random probability of being selected or not. At each turn, a subset of features is selected following the *tree* and *default* policies. The classification accuracy of the current feature subset is used as a reward, and the search tree is updated through backpropagation. The feature selection tree and four steps search procedure are graphically represented in Figure 3. Table 1 summarizes the notations used throughout the text.

### 4.1. Feature Subset Generation

A feature subset is generated during the *selection*, *expansion* and *simulation* steps, in each MCTS iteration.

#### 4.1.1. Selection

In the selection step, one path is selected from the already expanded tree. The path selected is the one whose inclusion gave a high reward in the previous iterations. The features in the selected path are included in the feature subset of the current iteration. The algorithm traverses the already expanded tree following the *tree* policy until a node is reached which is non-terminal and has an unexpanded child. The UCT algorithm is used to decide which node to be chosen at each level. If the UCT algorithm selects node $f_{i}$ at level i, feature $f_{i}$ is included in the current feature subset. If it selects $\emptyset_{i}$, feature $f_{i}$ is not included. If $f_{i}$ is selected, this is based on an intuition that the inclusion of feature $f_{i}$ gave a high reward in previous iterations, so needs to be included in the current feature subset. On the other hand, if it is not selected, it is better not to choose feature $f_{i\;}$ as it did not contribute much towards a better reward, previously.

The vanilla UCT algorithm approximates the reward at each node by dividing by the number of times the node is visited, as shown in Equation (1). This kind of approximation is most suitable in the game theoretic perspective where the reward is either a 1 (win) or 0 (loss), and the goal is to select the nodes (moves) which give the maximum number of wins in the minimum number of visits. However, feature selection is a different sort of a problem where the goal is to select the path which gives the maximum reward (accuracy). Using this intuition, instead of approximating the reward and penalizing by the number of visits, we used the maximum reward obtained at each node. The modified form of the UCT algorithm used in MOTiFS is shown in Equation (2). During tree traversal, the nodes which receive the highest scores from Equation (2) are selected until a non-terminal node with an unexpanded child is reached.

(2) $$UCT_{v_{j}}=\;\max\left(Q_{v_{j}}\right)+\;C\;\times \sqrt{\frac{2\;\times ln\left(N_{v_{i}}\right)}{N_{v_{j}}}},$$

where, $\max\left(Q_{v_{j}}\right)$ is the maximum reward at node $v_{j}$ and C>0 is a constant. $N_{v_{j}}$ and $N_{v_{i}}$ represent the number of times nodes $v_{j}$ and its parent $v_{i}$ are visited, respectively.

#### 4.1.2. Expansion

During expansion, a new child node is added to the urgent node (the last selected node in the selection step). The addition of a new child node at node $v_{i}$ is also based on the UCT function. If $UCT_{f_{i+1}}$ is larger than $UCT_{\emptyset_{i+1}}$ then child node $f_{i+1}$ is added, and thus, feature $f_{i+1}$ is included in the current feature subset. Conversely, child node $\emptyset_{i+1}$ is added and feature $f_{i+1}$ is not included in the current feature subset. 

#### 4.1.3. Simulation

The simulation step induces randomness in feature subset generation following the *default* policy. It choses features from the remaining unexpanded features, with a uniform probability of being selected or not. If the recently expanded node is $v_{i}$, a path from $v_{i}$ to a leaf node is randomly selected.

Assuming the current expanded node is $v_{i}$, the inclusion of features from $f_{1}$ to $f_{i}$ into the current feature subset is determined in the *selection* and *expansion* steps, whereas the inclusion of the remaining features from $f_{i+1}$ to $f_{n}$ in the current feature subset is randomly determined in the *simulation* step. A tree search and a random search participate together in feature subset generation, thus giving the opportunity to obtain the best feature subset in fewer runs even if the search tree is not fully expanded.

### 4.2. Reward Calculation and Backpropagation

The classifier is then applied to evaluate the goodness of the feature subset. The classification accuracy of the current feature subset is also used as a simulation reward, $Q_{simulation}$, for the current selected nodes and propagated backwards to update the search tree.

(3) $$Q_{simulation}=\;ACC_{classifier}\left(F_{subset}\right)$$

where, $ACC_{classifier}\left(.\right)$ represents the accuracy of the classifier on the current feature subset, $F_{subset}$. If the accuracy of the current feature subset is better than the previous best, then the current feature subset becomes the best feature subset. This process goes on until stopping criteria is met.

For the purpose of this study, we employed the nearest neighbors (*K-NN)* classifier to evaluate the candidate feature subset and as a reward function. We used the simple and efficient nearest neighbors classifier as it is well-understood in the literature and works surprisingly well in many situations [41,42,43,44]. Moreover, many other similar studies and comparison methods in literature, mentioned in Section 5.3, have applied the nearest neighbors classifier and therefore, we considered it to be a better choice for the comparative analysis. However, any other classifier can be used within the proposed framework. The algorithm for MOTiFS is provided below as Algorithm 1.

**Algorithm 1** The MOTiFS AlgorithmLoad dataset and preprocess Initialize SCALAR, BUDGET *//Scaling factor & Number of MCTS simulations (hyper parameters)* **function** MOTiFS (*featuresList*)  create *rootNode*  *maxReward, bestFeatureSubset* ← UCTSEARCH (*rootNode*)  **return** (*maxReward, bestFeatureSubset*) **function** UCTSEARCH (*rootNode*)  Initialize *maxReward, bestFeatureSubset*  **while** within computational budget **do**   *frontNode* ← TREEPOLICY (*rootNode*)   *reward, featureSubset* ← DEFAULTPOLICY (*frontNode.state*)   BACKUP (*frontNode, reward*)   **if**
*reward* is greater than *maxReward*
**then**    *maxReward* ← *reward*    *bestFeatureSubset* ← *featureSubset*  **return** (*maxReward, bestFeatureSubset*) **function** TREEPOLICY (*node*)  **while**
*node* is non-terminal **do**   **if**
*node* not fully expanded **then**    **return** EXPAND (*node*)   **else**    *node* ← BESTCHILD (*node, SCALAR*)  **return**
*node* **function** EXPAND (*node*)  choose *a*
∈ untried actions from *A*(*node.state*)  add a *newChild* with *f*(*node.state, a*)  **return**
*newChild* **function** BESTCHILD (v, C)  **return**
$\underset{v^{\prime }\in \;\mathrm{children}\;\mathrm{of}\;v}{\max}\max\left(Q_{v^{\prime }}\right)+C\sqrt{\frac{2\;\times \;ln\left(v.visits\right)}{v^{\prime }.visits}}$ **function** DEFAULTPOLICY (*state*)  **while**
*state* is non-terminal **do**   choose *a*
∈
*A*(*state*) uniformly at random  state ← *f*(*state, a*) **traverse**
*state.path*   **if**
*a_i_* is equal to *f_i+1_*
**then**    *featureSubset* ← INCLUDE (f_i+1_)   reward ← REWARD (*featureSubset*)   **return** (*reward, featureSubset*) **function** BACKUP (*node, reward*)  **while**
*node* is not null **do**   *node.visits* ← *node.visits* + 1   **if**
*reward* > *node.reward*
**then**    *node.reward* ← *reward*   *node* ← *node.parent*  **return**

## 5. Experiment and Results

The efficacy of the MOTiFS was demonstrated by experimenting on many publicly-available benchmark datasets. Twenty-five benchmark datasets of varying dimensions were used and results were compared with other significant methods in the literature.

### 5.1. Datasets

Twenty-five benchmark datasets were used for validation and comparison purposes. Twenty-four datasets were taken from two publicly available repositories, LIBSVM [45] and UCI [46]. However, one dataset “Klekota Roth fingerprint (KRFP)” representing the fingerprints of the chemical compounds was taken from the 5-HT_5A_ dataset to classify between active or inactive compounds [11,47]. The details of the datasets are summarized in Table 2. The datasets taken were of varying dimensions and sizes. In feature selection, literature categorizes datasets into three dimensional ranges, based on the total number of features (*F*): low dimension (0–19), medium dimension (20–49), and high dimension (50–∞) [48]. In the current study, 10 datasets were low dimensional, 5 datasets were medium dimensional, and 10 datasets were high dimensional.

### 5.2. Experimental Procedure and Parameter Setup for MOTiFS

We conducted 10-fold cross validation for the whole feature selection procedure. A dataset was equally divided into 10 random partitions. Then, a single partition was retained as a test set, while the remaining 9 partitions were used as a training set. This procedure was repeated 10 times (each partition behaved as a test set exactly once).

The significant advantage of MOTiFS is that only two hyper-parameters are required to be tuned. The parameter values used in the experiments are presented in Table 3. The “*scaling factor*”, *C*, maintains the balance between exploiting good solutions and exploring new ones in the search space. An excessively large value of *C* benefits the exploration part and slows down convergence, whereas, an insufficient *C* may cause the search to be stuck in a local optimum by penalizing the exploration and only sticking to locally good solutions. After careful examination and series of experimentation, we limited our choice of *C* to 0.1, 0.05, and 0.02. During training, we constructed three feature selection trees with different scaling factors (0.1, 0.05, 0.02) and selected one of them based on 5-fold cross validation accuracy. That is, the scaling factor was auto-tuned during the training process. For the “*termination criteria*”, we used the fixed *number of iterations* (*MCTS simulations)*. This depends on the dimensional size of the dataset. We set the number of simulations to 500 if the total number of features dimensions was less than 20, otherwise it was set to 1000, excluding the “KRFP” dataset. For the very high dimensional dataset, “KRFP”, we used 10,000 simulations.

### 5.3. Comparison Methods

We compared MOTiFS with state-of-the-art methods in the literature. The comparison methods were diverse and varied between established sequential approaches, fuzzy rule-based, evolutionary and entropy reduction-based methods, as summarized in Table 4. Most of the comparison methods were wrapper based, except FS-FS and FR-FS (well established filter-based methods) which we included for generalized comparison of accuracies achieved with different datasets earlier. For fair comparison against each dataset we deployed the classifier reported in available comparison methods. For datasets “Spambase”, “WBC”, “Ionosphere”, “Arrhythmia”, “Multiple features”, “Waveform”, “WBDC”, “Glass”, “Wine”, “Australian”, “German number”, “Zoo”, “Breast cancer”, “DNA”, “Vehicle”, “Sonar”, “Hillvalley”, “Musk 1”, “Splice” and “KRFP”, the used classifier was *5-NN*. For datasets “Soybean-small”, “Liver disorders”, “Credit”, “Tic-tac-toe” and “Libras movement”, the *3-NN* classifier is deployed.

### 5.4. Results and Comparisons

We conducted 10-fold cross-validation for the whole feature selection procedure, as detailed in Section 5.2. As our method is heuristic, we ran our algorithm five times on every datasets and reported the average of five runs. We reported the average accuracy and the number of features selected. Table 5 and Table 6 present a detailed summary of the results and comparisons with other methods. The bold values in each row indicate the best among all the methods.

In Table 5, results are reported for 20 datasets using *5-NN* as a classifier. Comparing classification accuracies, it is obvious that MOTiFS overall outperformed on 15 datasets, namely “Spambase”, “WBC”, “Ionosphere”, “Multiple features”, “WBDC”, “Glass”, “Wine”, “Australian”, “German number”, “Breast cancer”, “Vehicle”, “Sonar”, “Musk 1”, “Splice” and “KRFP”, compared to all other methods. Moreover, for two datasets, “Arrhythmia” and “Waveform”, MOTiFS ranked second among all the competitors. However, for three datasets, namely “Zoo”, “DNA” and “Hillvalley”, MOTiFS did not perform well.

Table 6 presents the result according to *3-NN* on five datasets. Clearly, MOTiFS outperformed on four datasets, namely “Liver-disorders”, “Credit”, “Tic-tac-toe” and “Libras movement” in terms of classification accuracy. However, on the “Soybean-small” dataset, the average MOTiFS score was not 1.0, as reported by all other methods, although MOTiFS achieved an accuracy of 1.0 on three out of five independent runs.

We also reported the standard deviation of five independent runs of MOTiFS for each dataset in Table 5 and Table 6, according to the average accuracy. For almost all of the datasets, the standard deviation was too small to be negligible. Thus, the stability and reliability of MOTiFS was evident.

**Figure 4:**
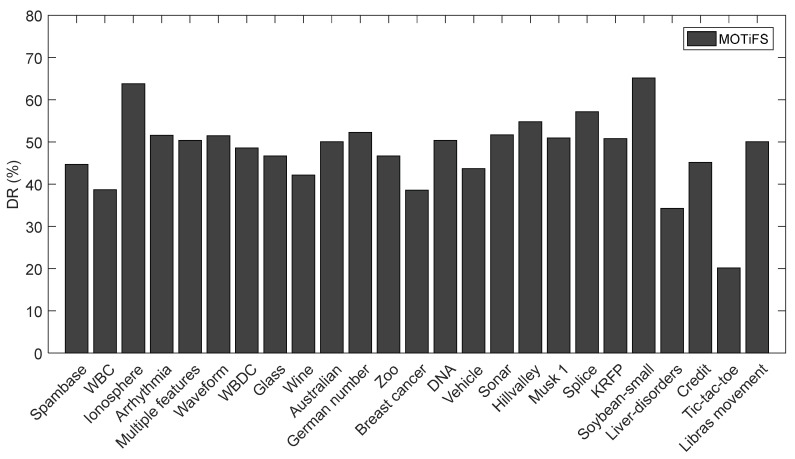
Graphical representation of dimensional reduction (DR) achieved by MOTiFS on all the datasets.

While comparing the number of selected features in Table 5, MOTiFS could not outperform because of marginal differences among all the methods. The reason is quite obvious; MOTiFS does not account for the selected features in reward evaluation and employs the classification accuracy only as the reward function. However, the DR (dimensional reduction) achieved by MOTiFS is presented in Figure 4. We calculate the DR on each dataset using Equation (4).

(4) $$\mathrm{DR}=1-\;\frac{\#\;selected\;features}{\#\;total\;features}$$

MOTiFS performed remarkably well in terms of accuracy and DR on all datasets compared to all other methods. On high dimensional datasets, “Ionosphere”, “Arrhythmia”, “Multiple features”, “Sonar”, “Musk 1”, “Splice”, “KRFP” and “Libras movement”, MOTiFS obtained DR values above 50%, achieving a high accuracy compared to other methods.

Summarizing the overall performance, MOTiFS outperformed the other methods on 19 out of 25 datasets, namely “Spambase”, “WBC”, “Ionosphere”, “Multiple features”, “WBDC”, “Glass”, “Wine”, “Australian”, “German number”, “Breast cancer”, “Vehicle”, “Sonar”, “Musk 1”, “Splice”, “KRFP”, “Liver-disorders”, “Credit”, “Tic-tac-toe” and “Libras movement”, compared to all other methods, in term of classification accuracy. For three datasets, “Arrhythmia”, “Waveform” and “Soybean-small”, MOTiFS ranked second among all methods. However, for three datasets, “Zoo”, “DNA” and “Hillvalley”, MOTiFS could not stand as high as the other methods. Overall, considering the accuracy and dimensional reduction together, MOTiFS performed remarkably well, as discussed above.

### 5.5. Discussion

We studied the effectiveness of MCTS in feature selection, which had previously been barely researched. Defining a feature selection problem as the proposed feature selection tree and applying MCTS to find best feature subset is a new concept, to the best of our knowledge. The proposed feature selection tree has the potential benefit of having less branching factors. A tree can grow to sufficient depth in a limited number of simulations, thus, taking adequate benefits from both the tree search and random sampling.

For the total number of features, n, if complexities in the node selection operation (UCT algorithm + random selection) and classifier are b and c, respectively, then the complexity of one MCTS simulation is O(nb+c). However, the complexity of the node selection operation is a constant, so the complexity for s number of simulations is O(sn+sc). If the number of simulations is fixed, the complexity of our proposed method is linear to the number of features, excluding the complexity of the classifier. Our proposed method finds the best feature subset within a limited number of simulations, as shown in the reported results and comparisons above.

We performed extensive experiments on 25 datasets of varying dimensions and sizes, with an aim of properly testing the performance of the proposed method. The results and comparisons with other state-of-the-art and evolutionary approaches showed the efficacy and usefulness of the proposed method. However, the performance could further be improved by careful examination of the datasets’ characteristics, modifying the reward functions and optimization of model parameters accordingly.

Future research directions may include experimentations on very large dimensional datasets and/or playing with different reward functions with an intention to further improve the performance in terms of both increasing accuracy and reducing dimensions.

## 6. Conclusions

In this paper, we proposed a novel feature selection algorithm, MOTiFS, which combines the robustness and dynamicity of MCTS with the accuracy of wrapper methods. MOTiFS searched the feature space efficiently by balancing between exploitation and exploration and found the best feature subset within a few iterations. Another significant feature of MOTiFS was the involvement of only two hyper-parameters: *scaling factor* and *termination criteria*, thus, making MOTiFS simple and flexible to handle. The *K*-NN classifier was used for experiments, and results were compared with the significant and state-of-the-art methods in the literature. Besides offering an improved classification accuracy on 25 real-world datasets, MOTiFS significantly reduced the dimensions of high dimensional datasets.

## Figures and Tables

**Table 1:** Notations used in the proposed method.

Notation	Interpretation
F	Original feature set
n	Total number of features
$v_{i}$	Node v at tree level i
$a\left(v_{i}\right)$	Action taken at $v_{i}$
$Q_{\mathrm{simulation}}$	Simulation reward

**Table 2:** Summary of the selected datasets.

#	Dataset	No. of Features	No. of Instances	No. of Classes
1	Spambase	57	4701	2
2	WBC	9	699	2
3	Ionosphere	34	351	2
4	Arrhythmia	195	452	16
5	Multiple features	649	2000	10
6	Waveform	40	5000	3
7	WBDC	30	569	2
8	Glass	9	214	6
9	Wine	13	178	3
10	Australian	14	690	2
11	German number	24	1000	2
12	Zoo	17	101	7
13	Breast cancer	10	683	2
14	DNA	180	2000	2
15	Vehicle	18	846	4
16	Sonar	60	208	2
17	Hillvalley	100	606	2
18	Musk 1	166	476	2
19	Splice	60	1000	2
20	KRFP *	4860	215	2
21	Soybean-small	35	47	4
22	Liver disorders	6	345	2
23	Credit	15	690	2
24	Tic-tac-toe	9	985	2
25	Libras movement	90	360	15

* downloaded from [11].

**Table 3:** Parameters setup for MOTiFS.

Parameter	Values Used for Different Datasets
Scaling factor, *C*	(0.1, 0.05, 0.02)
Termination criteria	(500, 1000, 10,000) iterations

**Table 4:** Summary of the methods for our comparison.

Method	Description
SFS, SBS	Sequential Forward Selection and Sequential Backward Selection * [22] (2015)
FS-FS	Feature Similarity Technique [49] (2002)
FR-FS	Fuzzy Rule Based Technique [50] (2012)
SFSW	An Evolutionary Multi-Objective Optimization Approach [22] (2015)
DEMOFS	Differential Evolution Based Multi-Objective Feature Selection * [22] (2014)
BA	Bat Algorithm and Optimum-Path Forest Based Wrapper Approach [26] (2014)
PSO	Particle Swarm Optimization Based Method * [26] (2014)
SCE, CCE	Shannon’s Entropy Reduction, Complementary Entropy Reduction * [39] (2016)
PDE-2	Partition Differential Entropy Based Method [39] (2016)

* Reported from the mentioned reference.

**Table 5:** Comparison of MOTiFS with other methods, according to *5-NN.*

Dataset	Avg. Acc. # Sel. Feat.								
	MOTiFS	SFSW [22]	SFS [22]	SBS [22]	FS-FS [49]	FR-FS [50]	DEMOFS [22]	BA [26]	PSO [26]
Spambase	**0.907** ± 0.003	0.885	0.874	0.870	0.900				
	31.5	**26.0**	35.7	37.3	29.0				
WBC	**0.968** ± 0.001	0.961	0.960	0.951	0.956				
	5.52	4.2	6.4	7.3	**4.0**				
Ionosphere	**0.889** ± 0.007	0.883	0.887	0.859	0.788	0.844		0.780	0.790
	12.32	11.5	**1.2**	9.1	16.0	4.33		21.0	14.0
Arrhythmia	0.650 ± 0.003	**0.658**	0.599	0.580	0.589				
	94.4	100.0	89.4	**49.2**	100.0				
Multiple features	**0.980** ± 0.001	0.979	0.903	0.912	0.783				
	321.84	270.0	**210.0**	305.0	325.0				
Waveform	0.816 ±0.002	**0.837**	0.778	0.785	0.752				
	19.42	**16.0**	18.4	18.3	20.0				
WBDC	**0.967** ± 0.004	0.941	0.901	0.898		0.936			
	15.42	13.5	13.9	17.8		**2.14**			
Glass	**0.705** ± 0.003	0.678	0.631	0.636		0.615			
	4.80	**4.4**	5.8	7.0		6.96			
Wine	**0.963** ± 0.004	0.961	0.914	0.914		0.955	0.897		
	7.52	6.9	6.0	7.5		**4.38**	6.0		
Australian	**0.850** ± 0.002	0.846	0.830	0.828			0.773		
	6.98	4.7	3.7	**3.0**			4.0		
German number	**0.725** ± 0.008	0.713	0.682	0.658			0.701		
	11.46	10.5	12.2	10.8			**1.0**		
Zoo	0.920 ± 0.022	0.954	0.949	**0.980**			0.954		
	9.06	11.0	**9.0**	13.0			11.0		
Breast cancer	**0.967** ± 0.003	0.965	0.951	0.949				0.940	0.930
	6.14	**4.3**	6.10	6.10				5.0	5.0
DNA	0.810 ± 0.006	**0.831**	0.822	0.823				0.760	0.760
	89.26	71.8	**18.8**	20.6				96.0	91.0
Vehicle	**0.721** ± 0.008	0.653	0.686	0.673					
	10.14	**9.1**	10.8	10.7					
Sonar	**0.850** ± 0.002	0.827				0.729	0.786		
	28.96	20.0				**5.85**	10.0		
Hillvalley	0.535 ± 0.003	0.575					**0.605**		
	45.18	40.0					**26.0**		
Musk 1	**0.852** ± 0.003	0.815					0.835		
	81.34	59.3					**58.0**		
Splice	**0.778** ± 0.002							0.680	0.670
	**25.66**							28.0	28.0
KRFP	**0.896** ± 0.001		0.842 *						0.884 *
	2390.2		**6.0**						1866

* evaluated using weka library.

**Table 6:** Comparison of MOTiFS with other methods, according to *3-NN.*

Dataset	Avg. Acc. # Sel. Feat.	Avg. Acc.		
	MOTiFS	SCE [39]	CCE [39]	PDE-2 [39]
Soybean-small	0.988 ± 0.015	**1.000**	**1.000**	**1.000**
	12.18			
Liver-disorders	**0.645** ± 0.012	0.602	0.592	0.590
	3.94			
Credit	**0.845** ± 0.007	0.646	0.654	0.659
	8.22			
Tic-tac-toe	**0.794** ± 0.006	0.774	0.747	0.757
	7.18			
Libras movement	**0.807** ± 0.011	0.538	0.552	0.554
	44.94			

## References

[1] Yu L., Liu H. (2004). Efficient Feature Selection via Analysis of Relevance and Redundancy. J. Mach. Learn. Res..

[2] Gasca E., Sánchez J.S., Alonso R. (2006). Eliminating redundancy and irrelevance using a new MLP-based feature selection method. Pattern Recognit..

[3] Gheyas I.A., Smith L.S. (2010). Feature subset selection in large dimensionality domains. Pattern Recognit..

[4] Zheng Y., Kwoh C.K. (2011). A Feature Subset Selection Method Based On High-Dimensional Mutual Information. Entropy.

[5] Robnik-Šikonja M., Kononenko I. (2003). Theoretical and Empirical Analysis of ReliefF and RReliefF. Mach. Learn..

[6] Sluga D., Lotrič U. (2017). Quadratic mutual information feature selection. Entropy.

[7] Hastie T., Tibshirani R., Friedman J. (2009). The Elements of Statistical Learning. Elements.

[8] Guo Y., Berman M., Gao J. (2014). Group subset selection for linear regression. Comput. Stat. Data Anal..

[9] Saganowski S., Gliwa B., Bródka P., Zygmunt A., Kazienko P., Kozlak J. (2015). Predicting community evolution in social networks. Entropy.

[10] Reif M., Shafait F. (2014). Efficient feature size reduction via predictive forward selection. Pattern Recognit..

[11] Śmieja M., Warszycki D. (2016). Average information content maximization-a new approach for fingerprint hybridization and reduction. PLoS ONE.

[12] Dash M., Choi K., Scheuermann P., Liu H. Feature selection for clustering-a filter solution. Proceedings of the 2002 IEEE International Conference on Data Mining, ICDM 2003.

[13] Kim Y., Street W.N., Menczer F. Feature selection in unsupervised learning via evolutionary search. Proceedings of the 6th ACM SIGKDD International Conference on Knowledge Discovery and Data Mining.

[14] Xue B., Zhang M., Browne W.N., Yao X. (2016). A Survey on Evolutionary Computation Approaches to Feature Selection. IEEE Trans. Evol. Comput..

[15] Hamdani T.M., Won J.M., Alimi A.M., Karray F. (2011). Hierarchical genetic algorithm with new evaluation function and bi-coded representation for the selection of features considering their confidence rate. Appl. Soft Comput. J..

[16] Hong J.H., Cho S.B. (2006). Efficient huge-scale feature selection with speciated genetic algorithm. Pattern Recognit. Lett..

[17] Kabir M.M., Shahjahan M., Murase K. (2012). A new hybrid ant colony optimization algorithm for feature selection. Expert Syst. Appl..

[18] Tabakhi S., Moradi P. (2015). Relevance-redundancy feature selection based on ant colony optimization. Pattern Recognit..

[19] Unler A., Murat A., Chinnam R.B. (2011). Mr2PSO: A maximum relevance minimum redundancy feature selection method based on swarm intelligence for support vector machine classification. Inf. Sci..

[20] Zhang Y., Gong D., Hu Y., Zhang W. (2015). Feature selection algorithm based on bare bones particle swarm optimization. Neurocomputing.

[21] Xue B., Zhang M., Browne W.N. (2012). Single feature ranking and binary particle swarm optimisation based feature subset ranking for feature selection. Conf. Res. Pract. Inf. Technol. Ser..

[22] Paul S., Das S. (2015). Simultaneous feature selection and weighting—An evolutionary multi-objective optimization approach. Pattern Recognit. Lett..

[23] Cordon O., Herrera F., del Jesus M.J., Villar P. A multiobjective genetic algorithm for feature selection and granularity learning in fuzzy-rule based classification systems. Proceedings of the IFSA World Congress and 20th NAFIPS International Conference.

[24] Xue B., Fu W., Zhang M. Multi-objective Feature Selection in Classification: A Differential Evolution Approach. Proceedings of the 10th International Conference on Simulated Evolution and Learning.

[25] Nakamura R.Y.M., Pereira L.A.M., Costa K.A., Rodrigues D., Papa J.P., Yang X.S. BBA: A binary bat algorithm for feature selection. Proceedings of the Brazilian Symposium of Computer Graphic and Image Processing.

[26] Rodrigues D., Pereira L.A.M., Nakamura R.Y.M., Costa K.A.P., Yang X., Souza A.N., Papa J.P. (2014). A wrapper approach for feature selection based on Bat Algorithm and Optimum-Path Forest. Expert Syst. Appl..

[27] Montazeri M. (2016). HHFS: Hyper-heuristic feature selection. Intell. Data Anal..

[28] Browne C., Powley E. (2012). A survey of monte carlo tree search methods. IEEE Trans. Intell. AI Games.

[29] Silver D., Huang A., Maddison C.J., Guez A., Sifre L., van den Driessche G., Schrittwieser J., Antonoglou I., Panneershelvam V., Lanctot M. (2016). Mastering the game of Go with deep neural networks and tree search. Nature.

[30] Hall M. (1999). Correlation-based Feature Selection for Machine Learning. Methodology.

[31] Senawi A., Wei H.L., Billings S.A. (2017). A new maximum relevance-minimum multicollinearity (MRmMC) method for feature selection and ranking. Pattern Recognit..

[32] Zhao G.D., Wu Y., Chen F.Q., Zhang J.M., Bai J. (2015). Effective feature selection using feature vector graph for classification. Neurocomputing.

[33] Huang C.L., Wang C.J. (2006). A GA-based feature selection and parameters optimizationfor support vector machines. Expert Syst. Appl..

[34] Durbha S.S., King R.L., Younan N.H. (2010). Wrapper-based feature subset selection for rapid image information mining. IEEE Geosci. Remote Sens. Lett..

[35] Kohavi R., John G.H. (1997). Wrappers for feature subset selection. Artif. Intell..

[36] Bermejo P., Gámez J.A., Puerta J.M. (2011). A GRASP algorithm for fast hybrid (filter-wrapper) feature subset selection in high-dimensional datasets. Pattern Recognit. Lett..

[37] Solorio-Fernández S., Carrasco-Ochoa J.A., Martínez-Trinidad J.F. (2016). A new hybrid filter–wrapper feature selection method for clustering based on ranking. Neurocomputing.

[38] Almuallim H., Dietterich T.G. (1994). Learning Boolean concepts in the presence of many irrelevant features. Artif. Intell..

[39] Li F., Zhang Z., Jin C. (2016). Feature selection with partition differentiation entropy for large-scale data sets. Inf. Sci..

[40] Gaudel R., Sebag M. Feature Selection as a One-Player Game. Proceedings of the International Conference on Machine Learning.

[41] Szenkovits A., Meszlenyi R., Buza K., Gasko N., Lung R.I., Suciu M. (2018). Feature Selection with a Genetic Algorithm for Classification of Brain Imaging Data.

[42] Buza K., Alexandros N., Lars S.-T. Time-series classification based on individualized error prediction. Proceedings of the IEEE 13th International conference on Computational Science and Engineering (CSE).

[43] Chen G.H., Stanislav N., Devavrat S. (2013). A latent source model for nonparametric time series classification. Advances in Neural Information Processing Systems.

[44] Devroye L., Gyorfi L., Krzyzak A., Lugosi G. (1994). On the Strong Universal Consistency of Nearest Neighbor Regression Function Estimates. Ann. Stat..

[45] Chang C., Lin C. (2001). Retrieved from LIBSVM—A Library for Support Vector Machines. https://www.csie.ntu.edu.tw/~cjlin/libsvm/.

[46] Machine Learning Repository Retrieved from University of California, Irvine. http://archive.ics.uci.edu/ml/index.php.

[47] Klekota J., Roth F.P. (2008). Chemical substructures that enrich for biological activity. Bioinformatics.

[48] Tahir M.A., Bouridane A., Kurugollu F. (2007). Simultaneous feature selection and feature weighting using Hybrid Tabu Search/K-nearest neighbor classifier. Pattern Recognit. Lett..

[49] Mitra P., Murthy C.A., Pal S.K. (2002). Unsupervised feature selection using feature similarity. IEEE Trans. Pattern Anal. Mach. Intell..

[50] Chen Y.-C., Pal N.R., Chung I.-F. (2012). An Integrated Mechanism for Feature Selection and Fuzzy Rule Extraction for Classification. IEEE Trans. Fuzzy Syst..

